# COVID-19 vaccine development, production and regulatory oversight in African countries

**DOI:** 10.2471/BLT.22.287958

**Published:** 2022-09-02

**Authors:** Geofrey Makenga, Robert Booy, Paul Ndaya Oloo, Joachim Auerbach

**Affiliations:** a National Institute for Medical Research, Tanga Centre, PO Box 5004, Hospital street, Tanga 255, Tanzania.; b Faculty of Medicine and Health, University of Sydney, Sydney, Australia.; c Coalition for Epidemic Preparedness Innovations, London, England.

According to the Africa Centres for Disease Control and Prevention (CDC), until 3 July 2022, Africa has had 11.8 million coronavirus disease 2019 (COVID-19) confirmed cases and 254 179 COVID-19 deaths. However, while three quarters of COVID-19 vaccine doses supplied had been used, only less than a quarter of the African population has been fully vaccinated. New pandemic waves driven by the regular emergence of new variants complicates the situation. The African Union has a population of 1.2 billion and 55 Member States, but imports almost all of its vaccines and consumes one quarter of the global vaccine supply.^1^ Apart from yellow fever vaccine production by the Institut Pasteur de Dakar in Senegal, no other World Health Organization (WHO) prequalified vaccine manufacturer exists in Africa. Although a few fill and finish pharmaceutical companies just fill the vaccine obtained from other manufacturers into vials, label and package,^1^^,^^2^ no capacity for active ingredient manufacture and even for fill and finish is available. The existing capacity falls short of guaranteeing self-sufficiency, especially in the current dynamic pandemic situation. 

Handling of the current and previous pandemics such as the 2009–2010 H1N1 influenza pandemic and the 2013–2016 Ebola virus disease outbreak should teach us how to better prepare for future pandemics.^3^ In search of how investment for vaccine production in Africa could be triggered, a study was published before the COVID-19 outbreak on how Africa’s local production could fit well in a broad business model that would ensure sustainable new vaccine introduction.^4^ Indeed, the COVID-19 pandemic has served as a wake-up call for most African countries and the pan-African or regional communities to venture into vaccine production.

Several vaccine production scenarios have been widely discussed in Africa, through either the Africa CDC or other stakeholders such as the African Union Development Agency-New Partnership for Africa’s Development. At least six countries have declared an interest in pursuing the vaccine production pathway, including some by fill and finish or distribution only and others through training their personnel. Several of these declarations were triggered by the intent of vaccine manufacturers such as Pfizer/BioNTech and Moderna to invest in Africa. However, the decision in most of these countries has been based on either a single country-restricted approach or a political move for public support. Importantly, the long-term success of these decisions may not be sustainable due to a potential difficulty in achieving a return on investment. Uncoordinated technology transfer to a single country and establishment of vaccine manufacturing approaches will likely not favour sustainable manufacturing capacity-building in Africa, leading countries to compete in the same vaccine market. Thus, uncoordinated vaccine production initiatives driven by national (single-country) approaches may reduce the sustainability of a business model and jeopardize market access.

Despite delays in vaccination, the COVID-19 death toll measured in Africa has been lower than in other continents.^5^ WHO foresees that COVID-19 related deaths in Africa may fall by over three quarters in 2022 thanks to vaccination, other nonpharmaceutical control measures and natural immunity.^6^ Therefore, a shift in political focus to other ongoing or emerging crises such as the rise in the cost of living may affect the pandemic-induced political commitment and previously declared priorities to establish local COVID-19 vaccine production. Unless countries in pursuit of local vaccine production intend to widen the scope beyond the current pandemic in a coordinated approach to produce other vaccines in addition to COVID-19 vaccines, most national vaccine production facilities will not succeed because of the high maintenance cost.

For a business model to be sustained, economies of scale have to be attained and maintained; a market based on a single country has less likelihood to guarantee this. Thus, a regional approach could bring these efforts to sustainable success. In the event that countries succeed in establishing their own vaccine production facilities, they should at least agree to specialize or share production and procurement of critical materials such as active product ingredients and technology know-how. Cooperation with other countries in the same economic zone would be a good start.

If the African Medicines Agency Member States or the countries of any regional economic zone agreed on a vision to enable sustainable local vaccine manufacture and respective regulatory oversight, advanced marketing commitments – that is, purchasing agreements – for the locally produced vaccines could provide planning security for the collaborating countries’ vaccination programmes and the manufacturers’ business plans. During the African Union Development Agency-New Partnership for Africa’s Development COVID-19 related webinar series^7^ in 2020, the African Development Bank designed a financing mechanism with financing local manufacturers as the bank’s priority. The funding of the facility could thus be ensured by the investor (whether private or public) for example in collaboration with the African Development Bank, local government contributions and international grants. Sustainability would be guaranteed by the advanced purchasing agreements and local government incentives. 

According to WHO’s COVID-19 vaccine tracker, as of 14 June 2022, 166 COVID-19 vaccines in clinical development and 198 in preclinical development worldwide existed, but none or very little of this development was ongoing in Africa. Given the global emergence of severe acute respiratory syndrome coronavirus 2 variants, vaccine effectiveness trials in the African continent would enable confidence in the current vaccines. The shifts of the pandemic are clearly variant-driven; therefore, involving African research institutions in the development of COVID-19 vaccines against predominant variants in Africa – or broadly protective coronavirus vaccines – is important.^8^^,^^9^ Ideally, vaccine batches from an African manufacturing hub using good manufacturing practices should be tested in local clinical trials. Such ambition would likely quicken the capacity-building and technical transfer prospects for vaccines.

Technical transfer, local good manufacturing practice production and clinical development require regulatory oversight by local national regulatory authorities. At early stages, this oversight could be performed with the support of international partner agencies and would contribute to strengthening local regulatory agencies. Such day-to-day experience would lead to the development of strong African regulatory authorities including the African Medicines Agency. In addition, local production would trigger the need for developing the capability to assess applications for primary licensure, which would also enable early access to vaccines on the African continent.

Advanced marketing commitments in collaboration with Gavi, the Vaccine Alliance, through the United Nations Children’s Fund or the newly established African Vaccine Acquisition Trust (which procures COVID-19 vaccines on behalf of African Union Member States) would be another pillar for sustainable success. For example, advance purchase agreements by the African Vaccine Acquisition Trust helped secure a deal for fill and finish of the Janssen (Johnson & Johnson: Ad26.COV2.S) COVID-19 vaccine by Aspen (a multinational South African pharmaceutical company). However, given the current situation of the pandemic with a less virulent strain, the decrease in orders of COVID-19 vaccine and the growing global oversupply of vaccines flags a question on sustainability of the local vaccine production at Aspen – in the context of COVID-19 vaccine supply only. Therefore, the African Vaccine Acquisition Trust should consider the whole African vaccine market, especially as more countries will soon graduate from Gavi’s support. The Africa CDC estimates that Africa’s existing vaccine market is worth 1.3 billion United States dollars (US$), and this figure is expected to grow to about US$ 2.4 billion by 2030.^1^

WHO has devised an important opportunity for Africa through the establishment of a COVID-19 messenger ribonucleic acid (mRNA) vaccines technology transfer hub, based at Afrigen, South Africa, to scale up global manufacturing.^10^ Through this initiative, WHO is set to transfer a comprehensive technology package with possibilities to expand to other technologies in the future. Of the few countries that have applied to the hub, South Africa is currently the main player. The hub at Afrigen launched in July 2021 is fully ready to operate. With the main equipment in place, the hub has already produced its first batches of COVID-19 mRNA vaccines, marking a successful achievement in such a short time. WHO has offered this opportunity to Africa and other regions and has selected several beneficiaries in all regions to receive technology transfer on mRNA vaccines.^10^ WHO had provided similar assistance during the smallpox pandemic and had supported countries such as Brazil, Cuba and India in their local production development, especially on the vaccine good manufacturing practice regulatory aspect. We should expect the Africa CDC to explore this opportunity via the Partnerships for African Vaccine Manufacturing.^11^ The creation of an African vaccines procurement pooling mechanism and a vaccine manufacturing Deal Preparation Facility with supportive fundraising for ecosystem enablers (that is, frontrunner projects for vaccine manufacturing capacity-building) could be the key for success of the creation of a sustainable vaccine manufacturing landscape in Africa. The Partnerships for African Vaccine Manufacturing should cooperate with WHO’s technology transfer hubs to help in establishing the planned capability and capacity centres. 

The latest developments in Africa, with the ongoing operationalization of African Medicines Agency, the strong coordination efforts of the Africa CDC and the implementation of the Partnerships for African Vaccine Manufacturing gives grounds for optimism and hope that the vaccine manufacturing capacity and related regulatory capabilities can be achieved earlier than experts expected before the pandemic.
